# Microfluidically Assisted Synthesis of Calcium Carbonate Submicron Particles with Improved Loading Properties

**DOI:** 10.3390/mi15010016

**Published:** 2023-12-21

**Authors:** Alexey V. Ermakov, Sergei V. Chapek, Ekaterina V. Lengert, Petr V. Konarev, Vladimir V. Volkov, Vladimir V. Artemov, Mikhail A. Soldatov, Daria B. Trushina

**Affiliations:** 1 Institute of Molecular Theranostics, First Moscow State Medical University, 119991 Moscow, Russia; lengertkatrin@mail.ru (E.V.L.); trushina.d@mail.ru (D.B.T.); 2 The Smart Materials Research Institute, Southern Federal University, Sladkova 178/24, 344090 Rostov-on-Don, Russia; chapek@sfedu.ru (S.V.C.); mikhailsoldatov@sfedu.ru (M.A.S.); 3 Federal Scientific Research Centre “Crystallography and Photonics”, Russian Academy of Sciences, 119333 Moscow, Russia; peter_konarev@mail.ru (P.V.K.); volkicras@mail.ru (V.V.V.); artemov@crys.ras.ru (V.V.A.)

**Keywords:** advanced synthesis methods, nano- and microparticles, biomedicine, microfluidic chip, submicron calcium carbonate, particle morphology, drug delivery applications

## Abstract

The development of advanced methods for the synthesis of nano- and microparticles in the field of biomedicine is of high interest due to a range of reasons. The current synthesis methods may have limitations in terms of efficiency, scalability, and uniformity of the particles. Here, we investigate the synthesis of submicron calcium carbonate using a microfluidic chip with a T-shaped oil supply for droplet-based synthesis to facilitate control over the formation of submicron calcium carbonate particles. The design of the chip allowed for the precise manipulation of reaction parameters, resulting in improved porosity while maintaining an efficient synthesis rate. The pore size distribution within calcium carbonate particles was estimated via small-angle X-ray scattering. This study showed that the high porosity and reduced size of the particles facilitated the higher loading of a model peptide: 16 vs. 9 mass.% for the particles synthesized in a microfluidic device and in bulk, correspondingly. The biosafety of the developed particles in the concentration range of 0.08–0.8 mg per plate was established by the results of the cytotoxicity study using mouse fibroblasts. This innovative approach of microfluidically assisted synthesis provides a promising avenue for future research in the field of particle synthesis and drug delivery systems.

## 1. Introduction

In recent years, remarkable strides have been made in the advancement of methodologies for synthesizing materials intended for drug delivery systems [1,2,3,4]. At the forefront of this progress is the growing demand for the fabrication of drug delivery carriers that possess multifunctionality that, in turn, demand a specific structure, including core–shell structures and multi-component configurations. The biomedical challenges at hand necessitate a breakthrough in the development of complex and multi-component carriers with exceptional reproducibility and homogeneity. Such particles can provide a number of functions at once, including prolonged circulation in the blood, enhanced EPR effect, specific internalization, etc. [5,6,7]. These crucial advancements are necessary to enable efficient therapy even at the single-cell level [8,9,10]. 

One promising approach to improve synthesis procedures lies in the application of microfluidic technology, which opens up new avenues for the synthesis and analysis of advanced nanostructured materials [11,12]. The reason is the ability to unify synthesis conditions, which is often impossible to achieve in bulk, while simultaneously performing in situ analysis using different methods. The emergence of interdisciplinary technologies based on microfluidics has revolutionized the lab-on-chip approach, offering a unique means of producing nano- and microparticles [13,14]. The T-shaped design of the microfluidic chip allows for the formation of uniform droplets, maximizing the surface-to-volume ratio for efficient reaction kinetics. This feature ensures rapid mass transfer in microreactors (droplets) without the influence of reaction components on each other [15]. Furthermore, the microfluidic approach can enable more reproducible synthesis of porous inorganic particles and allow in situ analysis of the crystallization process using a range of techniques [4]. 

Among the materials garnering significant attention in microfluidic synthesis and study, calcium carbonate (CaCO_3_) stands as a potential drug carrier in a variety of tasks ranging from transdermal delivery to cancer therapy [16]. In the realm of biomedicine, calcium carbonate’s unique mesoporous structure, stiffness, and excellent biocompatibility have made it an exceptional candidate for innovative therapeutic interventions. These properties of calcium carbonate are based on the process of its synthesis, which consists of the incorporation of a large number of nuclei, resulting in the formation of a porous structure. During the CaCO_3_ crystallization, amorphous CaCO_3_ transforms into vaterite and subsequently into calcite. This transformation occurs through the Ostwald ripening process and crystal growth, which replaces the spherulitic growth of vaterite crystals. The ripening of vaterite crystals leads to the formation of calcite via a dissolution–precipitation process. This transformation is primarily governed by thermodynamics, as under normal conditions, vaterite particles tend to transition into the more thermodynamically stable calcite form within aqueous solutions. This natural transition occurs over time as solution-mediated recrystallization provides kinetic pathways for the transformation of vaterite crystals into more energetically favorable forms of calcium carbonate [17,18]. By manipulating synthetic conditions, including pH, reagent ratio, temperature, viscosity of the reaction volume, and the incorporation of organic and inorganic additives, one can exert control over the crystallinity, size, shape, and porosity of the resulting CaCO_3_ particles. Furthermore, the synthesis of CaCO_3_ particles comprising various phases, such as vaterite, calcite, and aragonite, with precise control over size and morphology is achievable without the use of additives via a vortex fluidic device [19,20,21,22]. Increasing the ability to finely tune the conditions of particle formation and reducing the resource to limit the formation of calcium carbonate crystal nuclei represents a promising strategy for controlling particle size and porosity and is currently often solved by the use of viscous media such as ethylene glycol and glycerol [23,24]. While the production of micro-sized calcium carbonate is relatively simple and efficient, producing particles of submicron size requires extensive time investment. In a typical synthesis conducted in a viscous medium, a meager 0.5–1 mL of salts with a concentration ranging from 0.3 to 1 M is introduced into 10–15 mL of the medium, resulting in a yield of approximately 5–20 mg of particles. Additionally, this method entails repetitive centrifugation steps to cleanse the medium molecules, further complicating an already intricate procedure that yields poor outputs [25,26]. Thus, calcium carbonate showcases a captivating display of complex polymorphic behavior, with each of its polymorphic modifications boasting unique characteristics that dictate its application. Among the polymorphic modifications that calcium carbonate can assume, polycrystalline vaterite in the form of mesoporous aggregates of submicron particles holds particular promise. 

In the realm of drug delivery, calcium carbonate in the form of vaterite emerges as a promising carrier owing to its remarkable porosity, exceptional loading capacity, and capability to template the formation of polymeric shells [27]. Basically, this procedure involves applying one or more layers of polymers, polyelectrolytes, hydrogels, etc., to the surface of calcium carbonate, followed by the dissolution of the calcium carbonate core. Among the diverse polymorphic modifications that calcium carbonate can assume, polycrystalline vaterite in the form of mesoporous aggregates of submicron particles holds particular promise. Vaterite particles, ranging in size from submicrons to microns, have demonstrated a remarkable ability to accommodate high drug payloads while affording excellent biocompatibility and facilitating the safe long-term storage of loaded drugs [16,28]. These properties, together with an average size and spherical shape, are essential in terms of drug delivery and should be carefully considered. Numerous studies have demonstrated the promising attributes and successful preclinical outcomes of using calcium carbonate particles for drug delivery purposes [29,30]. However, several key factors, such as charge, loading capacity, and biocompatibility, significantly impact the efficacy of calcium carbonate as a drug carrier.

Although the synthesis of CaCO_3_ is a relatively straightforward process involving the agitation and aging of salt solutions, ensuring the reproducibility of crystallization and homogeneity of the resulting particles remains a challenge. The formation of these particles is contingent upon numerous factors that are difficult to control [31]. Moreover, most of these factors have not been thoroughly described or discussed in scientific publications. For instance, the size of the magnetic anchor used for stirring and its correlation with the vessel size in which the synthesis is carried out, the speed of centrifugation, and more apparent factors like pH and synthesis temperature are often overlooked. These factors can potentially result in not only alterations in the size and polymorphic composition of the resulting suspension but also changes in its shape. Accordingly, a comprehensive study of the impact of these factors on particle porosity has not been conducted. Certain studies demonstrate that the aspect ratio of calcium carbonate particles (ratio of the length of a particle to its width) and its anisotropic growth can be attained through adjustments in salt concentrations, solvent composition, and particle formation time [25]. However, even a simple synthesis involving the mixing of two salts in aqueous media can frequently yield oval particles, thereby complicating the standardization of calcium carbonate synthesis.

The loading capacity of particles determines the quantity of drug that can be loaded into the carriers, thus influencing dosage and therapeutic effectiveness. Additionally, the biocompatibility of calcium carbonate particles is of paramount importance to ensure they are well tolerated by the body and the absence of any adverse reactions. The loading of bioactive substances onto calcium carbonate particles requires the utilization of co-precipitation procedures, which typically exhibit an average efficiency of approximately 2–4 wt.% in loading low-molecular-weight compounds, polymers and even nanoparticles. To enhance loading effectiveness, controlling the porosity of CaCO_3_ is a crucial factor influenced by the temperature of crystallization under supersaturated conditions. This is due to the fact that spherulitic crystal growth is accompanied by the Ostwald ripening of the nanocrystallites [31]. Through this method, calcium carbonate has been successfully loaded with various functional species via co-precipitation [32], including magnetite and silver nanoparticles, enabling targeted and probed functionalities, as demonstrated by surface-enhanced Raman signals [33]. 

Moreover, recent advancements in CaCO_3_-based microcarriers have paved the way for the creation of systems for transdermal delivery of antimycotics [34], antimicrobials [35,36], multifunctional proteins [37], and enzymes [38] with a variety of applications ranging from transdermal delivery to cancer therapy [2]. Alongside the loading strategies for vaterite particles with functional cargos, the synthesis of core–shell structures has emerged as a captivating trend. Diverse CaCO_3_-templated microcarriers have been developed, including those utilizing calcium carbonate as a functional core [39] or hollow capsules with the potential to remove the core [40].

In this paper, we present a methodology employed to harness the potential of a droplet-based approach within a 3D-printed microfluidic device for the synthesis of calcium carbonate particles. By harnessing this innovative technique, we successfully facilitate the synthetic generation of calcium carbonate particles while simultaneously exerting control over their growth process. A primary focus of this investigation lies in the evaluation of these particles from an applicability perspective, specifically aiming to evaluate their potential utility as drug carriers. Traditional methods of particle synthesis often suffer from challenges associated with uncontrolled growth, leading to a broader size distribution and reduced uniformity. By leveraging the precise control offered by microfluidic platforms, we aim to overcome these limitations and achieve a unified process for synthesizing particles with a narrower size range. Previously, it was shown by different studies that this approach enables the synthesis of uniform calcium carbonate particles [41,42]. The droplet method within microfluidics facilitates the generation of highly uniform droplets, which serve as confined reaction environments for particle formation. This confinement enables rapid and controlled nucleation and growth, minimizing undesired particle growth. By exploring various operating parameters and optimizing conditions within the microfluidic setup, we anticipate achieving superior control over particle size growth, resulting in narrower size distributions and enhanced uniformity. This method demonstrated its potential to efficiently stabilize particle growth dynamics by limiting the available resources for synthesis. The precise regulation of crucial parameters such as reaction time, concentration, and pH levels allows for the fine-tuning of specific properties of the resulting calcium carbonate particles. 

## 2. Materials and Methods

### 2.1. Chemicals

Calcium chloride (CaCl_2_), sodium carbonate (Na_2_CO_3_), castor oil, ethylene glycol, bovine serum albumin (BSA, 66 kDa), tetramethylrhodamine (TRITC), isopropyl alcohol, hexane, ethanol, Dulbecco’s minimum essential medium (DMEM), fetal bovine serum (FBS), Alamar blue, and calcein-AM were purchased from Sigma Aldrich (St. Louis, MO, USA). Minimum essential medium (MEM), penicillin, streptomycin, trypsin, and trypan blue were purchased from Thermo Fisher Scientific (Waltham, MA, USA). Deionized water with specific resistivity higher than 18.2 MΩ cm^−1^ from a three-stage Milli-Q Plus 185 purification system was used in the experiments.

### 2.2. Design and Fabrication of the 3D-Printed Microfluidics Device

**Figure 1:**
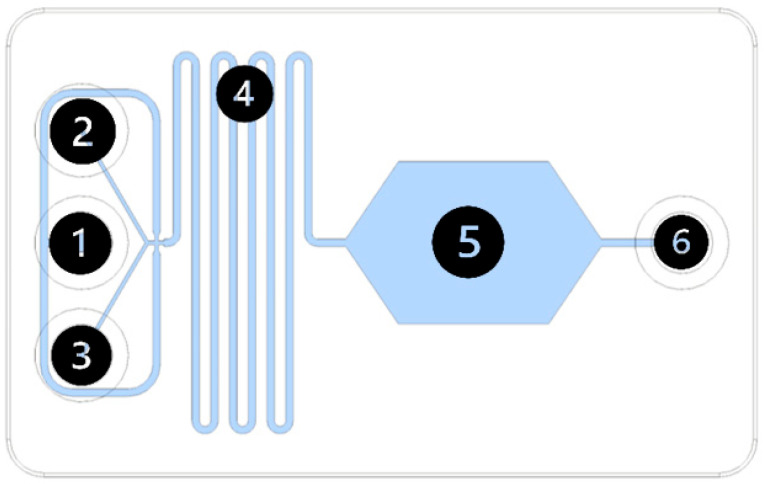
Topology of a droplet-based microfluidics device (1—transport phase inlet; 2—reagent 1; 3—reagent 2; 4—reaction zone; 5—droplet storage chamber; 6—outlet).

The topology of microfluidic device was designed using Fusion 360 CAD system (Autodesk, San Francisco, CA, USA) (the corresponding STL file can be found in the Supplementary Materials). Three-inlet design (Figure 1) has been chosen to form droplets in castor oil (inlet 1) from a mixture of two reagents (inlet 2–3). The channel cross-section was set to 200 µm × 200 µm before the flow focusing orifice and 400 µm × 200 µm in the reaction zone. A droplet storage chamber was used to monitor droplets’ shape and size. 

Microfluidic device was printed using digital light processing technology (DLP) implemented in MAX UV 3D printer (Asiga, Sydney, Australia) at the wavelength λ = 385 nm and a light intensity of 7.25 MW/cm^2^.

The protocols reported by Shapavalov et al. [43] were adapted to avoid delamination of the model from the platform. The following parameters were chosen: the height of the first layer—25 µm; the exposure time of the first layer—20 s. The subsequent thicknesses and exposure times of the layers were 25 µm and 800 ms, respectively. The printing photopolymer resin (FunToDo Nano Clear, Alkmaar, The Netherlands) temperature was set to 50 °C during the layer-forming process. The microfluidic device was sonicated in isopropyl alcohol for 60 s at 80 kHz and then placed in a holder for manual channel washing with the syringe immediately after printing. After the channels were washed, the devices were further sonicated and dried with compressed air. Finally, the chips were post-treated for 5 min using a UV light lamp (Flash DR-301C type, Asiga, Sydney, Australia).

### 2.3. Reaction of CaCO_3_ Synthesis

**Figure 2:**
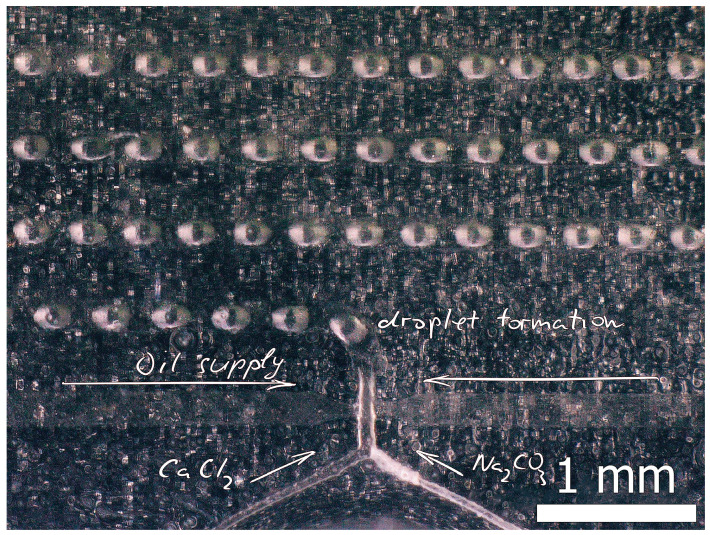
Microphotograph of the microfluidic device plane during formation of CaCl_2_/Na_2_CO_3_ solution droplets in castor oil.

Experimental samples were obtained in conditions of limited sources using droplet-based approach in a microfluidic device (Figure 2). To achieve the on-chip precipitation of calcium carbonate, we delivered small volumes of equimolar solutions of calcium chloride and sodium carbonate (0.5 M) through the two inlets of the microfluidic device at a flow rate of 0.5 mL/h. For the continuous flow experiments, this setup sufficed. However, for the generation of droplets, we used flow focusing of the aqueous stream with castor oil at a flow rate of 0.7 mL/h. The reaction products were collected at the outlet of the device’s tubing and transferred to 2 mL, followed by several cycles of washing with hexane and ethanol. After washing with ethanol under suction and air-drying, the resulting precipitates were obtained.

As a control sample, we performed synthesis of CaCO_3_ via traditional techniques employed in numerous studies. For this purpose, spherical CaCO_3_ microparticles with porous structures were synthesized following the methodology established by Volodkin et al. [44]. Briefly, a solution of 0.5 M CaCl_2_ (0.5 mL) and a solution of 0.5 M Na_2_CO_3_ (0.5 mL) were injected in a viscous medium comprised of 4 mL of ethylene glycol in a 25 mL laboratory glass, accompanied by agitation by a standard 1 cm long magnetic stirrer. Stirring this composition was performed at 500 rpm. The stirring was ceased after 2 h, enabling the dispersion of particles to settle. The ensuing dispersion was then separated through centrifugation and with two rinses of water.

### 2.4. Loading of CaCO_3_ Particles with Model Substances

The loading of CaCO_3_ microparticles was performed through the utilization of freezing-induced loading (FIL) through the following method reported previously [45]: a solution containing 2 mL of the BSA-TRITC solution was delicately introduced to 10 mg of CaCO_3_ particles. To facilitate the reaction, the mixture was carefully placed inside a microcentrifuge tube and transferred to a freezing chamber at a temperature of −17 °C for a duration of 2 h, all the while being stirred at a slow and constant pace. Subsequently, the samples were subjected to a thawing process at room temperature and thoroughly cleansed. Then, the samples were dried inside a drying cabinet. This freezing/thawing cycle was repeated 3 iterations.

In a different instance, the loading process was performed utilizing the co-precipitation technique, where the encapsulated substance was introduced alongside the synthesis of calcium carbonate particles. This loading method was implemented either within a microfluidic chip or in the bulk phase.

In order to perform the synthesis of the fluorescent conjugate to serve as a model substance, a solution of TRITC (1 mg) was meticulously dissolved in ethanol (5 mL). This resulting TRITC solution was subsequently introduced into a bovine serum albumin solution (20 mL, 4 mg/mL, carbonate–bicarbonate buffer, pH 8.5). The resultant mixture was diligently stirred for a duration of 12 h at a temperature of 4 °C. Following this step, the solution comprising TRITC conjugated with BSA was thoroughly cleansed of any leftover reagents through extensive dialysis in deionized water, a process which spanned a total of 4 days.

### 2.5. Characterisation

The precipitates collected and washed from both sets of experiments were examined using a range of methods. Samples were studied by scanning electron microscopy (SEM) using a JSM-7401F instrument (JEOL, Akishima, Japan). Secondary electron images were acquired using the high secondary electron detector at an accelerating voltage of 1 kV and Gentle Beam regime. To conduct SEM measurements, water suspension of calcium carbonate particles was deposited on a silicon wafer and dried.

X-ray diffraction (XRD) patterns were obtained using Rigaku Miniflex 600 (Bruker, Ettlingen, Germany) using CuKα radiation source (λ = 1.5406 Å, 40 kV, 15 mA). The XRD data were collected over a scanning range of 2θ from 11 to 70° and a step size of 0.02° 2θ. The phase composition was estimated from the XRD patterns after phase identification by using Rietveld analysis.

The concentrations of the model dye in the samples were assessed using spectroscopy measurements conducted with an Infinite 200 PRO microplate reader. The fluorescence intensities in the samples were quantified and compared to a pre-established calibration curve in order to determine the concentration of the model dye.

A confocal Raman microscope (Renishaw inVia, New Mills, UK) with 532 nm laser and power controlled by neutral optical density filters was used. 

The size distribution of the particle suspension was determined by averaging the data from 10 replicates using a Zetasizer Nano-Z instrument (Malvern Instruments Ltd., Malvern, UK).

The automatic small-angle X-ray scattering diffractometer “AMUR-K” (Institute of Crystallography, Moscow, Russia) equipped with a Kratky collimation system and an OD3M one-coordinate position-sensitive gas detector at a fixed wavelength λ = 0.1542 nm was employed for SAXS measurements. It provided the range of the momentum transfer 0.11 nm^−1^ < s < 10.0 nm^−1^ (where s = 4 πsinθ/λ, 2θ is the scattering angle, and λ is the wavelength). The studied powders of CaCO_3_ samples were measured in a specially designed 1 mm thick cuvette with mylar windows (15 μm thickness) that was placed in a vacuum chamber. The sample-to-detector distance was 700 mm, and the exposure time was 10 min. The correction for the collimation distortion was made according to the standard procedures [46]. The subtraction of the scattering signal of the empty cell from the sample scattering intensity was carried out with the program PRIMUS.

A number of approaches were used to calculate the pore size distributions under assumption of their spherical shapes:

(a) Direct search of pore size distribution by linear least squares method using indirect Fourier transformation with Tikhonov regularization of the solution (program GNOM [47] from the ATSAS package);
(b) Direct search of particle size distribution in the form of a histogram with an arbitrary shape (program VOLDIS);
(c) A parametric modeling of the pore distribution composed from a superposition of smooth analytical Schulz distribution functions (program MIXTURE from the ATSAS package). Non-linear minimization is employed to search the positions and half-widths of such functions and their relative contributions.

Each of the algorithms has some advantages and drawbacks, and only their combined use allows us to obtain reliable solutions. In particular, the distributions obtained using the program GNOM may exhibit significant oscillations due to the termination effects during Fourier transformations. In the program VOLDIS, the oscillations may come from the fact that the increase in the scattering contribution from particles of one radius is compensated by a decrease in the contribution from particles with nearby radii with only marginal changes in the scattering signal. The program MIXTURE is free of this drawback, but a successful search requires the starting values for the parameters of the partial distributions close enough to the global solution. Therefore, the distributions obtained with the GNOM and VOLDIS programs, were used as meaningful initial approximations for the program MIXTURE.

### 2.6. In Vitro Toxicity Assessment

Normal human dermal fibroblast (NHDF) cells were meticulously seeded in a 96-well cell culture plate at a density of 104 cells per well. Each well in the culture plate was thoughtfully filled with 100 μL of minimal essential medium (MEM) supplemented with 10% fetal bovine serum (FBS) and 1% penicillin–streptomycin. The plates were then meticulously incubated at a temperature of 37 °C in a 5% carbon dioxide (CO_2_) atmosphere. After a period of 24 h following the initial plating, varying dosages of calcium carbonate particles were incorporated into the culture medium, which was then subjected to overnight incubation. To reduce optical density, the cell culture medium was subsequently replaced with phosphate-buffered saline (PBS). In the final step, 10 μL of the fluorescence dye Alamar blue (10% *V*/*V*) was systematically added to each well to facilitate the detection of viable cells, followed by an additional 24 h of incubation. The fluorescence intensity of the samples, with an excitation wavelength of 560 nm and an emission wavelength of 590 nm, was then precisely measured using spectroscopy. The data obtained from cells cultured without the presence of any particles but with the corresponding volume of the water added to each sample were considered as the baseline with a viability of 100%.

## 3. Results

The optimization of microfluidic particle synthesis parameters was conducted through theoretical calculations and experimental tuning. The crude theoretical calculations were utilized to estimate the droplet size necessary for synthesizing submicron-sized particles under resource-constrained conditions. Additionally, experimental tuning of the parameters was carried out due to several unknown factors, such as the number of embryos in a single drop. Therefore, the focal point of the optimization process revolved around determining the ratio between the total flow rate of calcium chloride and sodium carbonate salts and castor oil, as it dictates the droplet size. Simultaneously, the total flow rate of the reaction mixture and oil does not hold great significance within the scope of this study, as it solely impacts the rate of particle synthesis. Nevertheless, in the absence of surfactant stabilizers, it is crucial to limit this rate due to the low stability of drops. Consequently, we experimentally determined the optimal ratio of water-soluble component speeds to oil speeds within the range of 1.3–1.5. High speeds of water-soluble components (as well as high salt concentrations) led to the production of larger particles, even reaching the micron range, whereas lower speeds of water-soluble components resulted in the formation of an aggregated mass without the ability to break into individual particles upon oil washing. Ultimately, the selected synthesis parameters prove sufficient for the production of nano- and submicron particles of calcium carbonate, successfully fulfilling the goal outlined in this study. However, it is important to note that these parameters may not be the sole accurate ones.

As a control in this study, submicron calcium carbonate particles were selected in accordance with a modified version of one of the most commonly employed protocols for synthesizing calcium carbonate particles [48,49,50,51]. Polyhydric alcohol was used as a medium to decelerate the reaction between calcium chloride and sodium carbonate. Due to the challenge of obtaining standardized data on porosity and polymorphic composition of calcium carbonate, the calcium carbonate produced using this protocol was employed as the control. Subsequently, the characteristics of the particles produced in the microfluidic device were compared against those of the control in order to assess their properties. 

**Figure 3:**
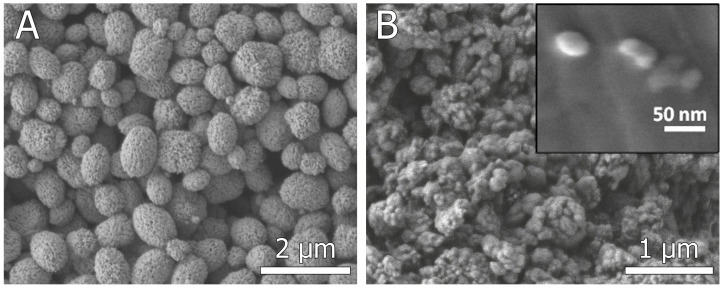
SEM images of CaCO_3_ particles synthesized in bulk conditions using ethylene glycol (**A**) and CaCO_3_ particles synthesized via droplet-based approach in a microfluidic chip (**B**).

**Figure 4:**
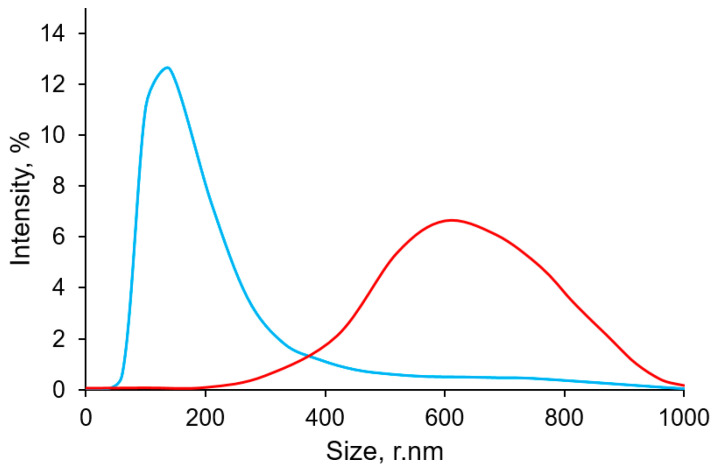
DLS results on the size distribution of CaCO_3_ particles synthesized in bulk conditions using ethylene glycol (red line) and CaCO_3_ particles synthesized via droplet-based approach in a microfluidic chip (blue line).

Obtained particles were collected, thoroughly washed, and analyzed. The SEM images depicted in Figure 3 illustrate the SEM of carbonate particles acquired from the microfluidic device alongside the distinctive SEM images of vaterite particles synthesized in ethylene glycol. Calcium carbonate particles synthesized under bulk conditions by adding ethylene glycol to salt solutions have a typical shape, almost spherical, with a developed surface, as clearly shown in the inset in Figure 3A. Such particles are known to be polycrystalline, consisting of individual nano-sized grains. Microfluidically assisted synthesis allowed for obtaining nano-sized spherical and slightly elongated calcium carbonate particles (Figure 3B). The minor agglomeration of nanoparticles may be due to the fact that synthetic low-molecular-weight surfactants are not used in the synthesis so as not to affect particle biocompatibility and ensure their low toxicity. Both the SEM and Dynamic Light Scattering (DLS) (Figure 4) outcomes manifest that the average dimension of the particles synthesized in the chip amounts to roughly 100 nm, yet they exhibit a notable degree of aggregation. Conversely, it is observed that the minimum size achievable through bulk synthesis is at least 350 nm with a peak around 600 nm.

Notably, DLS does not reveal the presence of large aggregates (over 800 nm) in the suspension synthesized using a microfluidic device. It is probable that the ultrasound treatment prior to the measurement effectively breaks up these aggregates, and aggregation in a relatively stable suspension occurs again only within an hour as the suspension precipitates. However, upon drying for SEM measurement, the increase in particle concentration leads to their aggregation once more. Nevertheless, the smallest particles with a size of 20 nm are also not detected by DLS measurements and appear to be present in the form of aggregates. 

Quantifying calcium carbonate polymorphs via XRD is a challenging task due to the absence of a valid structural model for elucidating the diffraction pattern of vaterite. The vaterite crystal structure has confounded scientists for nearly a hundred years, and several structural models have been proposed [52,53]. The series of peaks observed in the diffraction patterns obtained (Figure S2) can be most accurately explained by the hexagonal P63/mmc crystal structure with unit pseudocell parameters a = b = 4.13 Å, and c = 8.49 Å, as provided by Kamhi [54]. In this crystal structure, the carbonate groups are randomly distributed among three orientations. Calcite polymorph belongs to the trigonal $\bar{R}$ 3c space group, where Ca^2^⁺ is bonded to six equivalent O^2^⁻ atoms, forming corner-sharing CaO_6_ octahedra. Quantitative phase analysis was performed by a full profile analysis using the Rietveld method, utilizing the crystal structures presented (Figure S2). Powder X-ray diffraction analysis results indicate that microfluidic synthesis yields an almost single-phase nanoparticle composition containing mainly vaterite (96.5%) and a small fraction of calcite (3.5%). Submicron particles obtained as a result of bulk synthesis have a comparatively lower vaterite content, comprising 87.3% vaterite and 12.7% calcite.

Furthermore, the polymorphic composition of the obtained samples was evaluated using Raman spectroscopy (Figure S1). Subsequently, Raman microscopy of individual particles and mapping over an area of 100 μm^2^ was employed to further confirm these polymorph assignments. Calcite was distinguished by the presence of characteristic peaks at 711 cm^−1^ and 1085 cm^−1^, while vaterite exhibited a distinct doublet peak at 1088 cm^−1^. Interestingly, according to the Raman spectroscopy results, the samples synthesized within the microfluidic chip demonstrated a significantly lower proportion of calcite, with the vaterite fraction predominating. However, XRD analysis (Figure S2) detected approximately 12% calcite fraction in the samples obtained with the microfluidic device, while the control sample showed only 3%. On the one hand, this difference is close to the margin of error and sample variation, yet since Raman microscopy primarily examines the surface, and XRD has greater penetration depth, this may reflect differences in the structure of the resulting particles.

**Figure 5:**
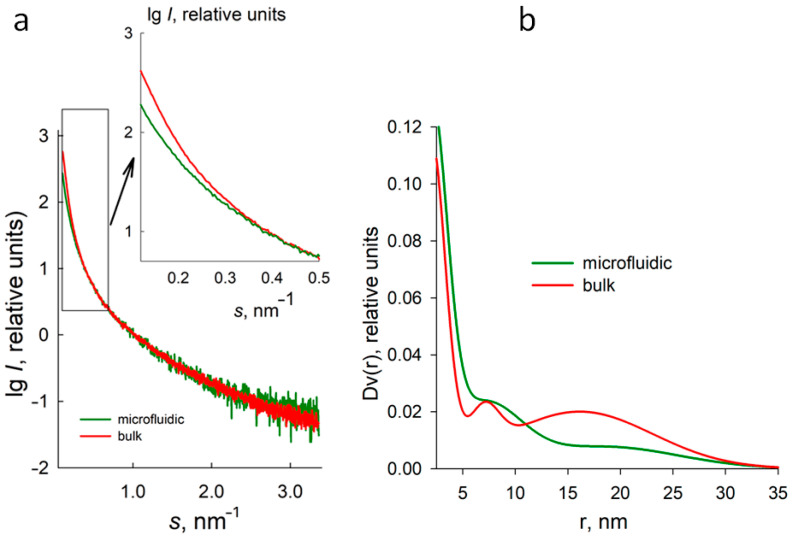
Experimental SAXS curves (**a**) and functions of pore size distributions Dv(r) assuming the spherical shapes of the voids (**b**) for CaCO_3_ in the bulk phase and synthesized within the microfluidic chip.

The obtained samples were analyzed using the small-angle X-ray scattering (SAXS) method (Figure 5). Within the bulk phase, the submicron vaterite particles demonstrated distributions of pore size radii within 3 and 33 nm. Interestingly, the vaterite particles synthesized within the microfluidic chip exhibited significantly smaller relative fractions of pores with radii ranging from 12 to 30 nm compared to the bulk phase (Figure 5b). This corresponds to the observed sharper decrease in scattering intensity from the bulk phase at very low angles (s < 0.5 nm^−1^) compared to those synthesized within the microfluidic chip (Figure 5a, inset). Comparing the two functions of pore size distributions in Figure 5b leads to the conclusion that despite the difference in particle sizes formed in bulk and microfluidic device, they both have nano-sized pores, as evidenced by the peak position in the range of 5–10 nm. However, the porosity of vaterite particles precipitated in bulk and microfluidically assisted synthesis differs in the range of 12–23 nm: the microfluidically generated vaterite particles practically have no pores in this range, unlike the other sample. These findings suggest that microfluidically assisted synthesis influences the structural characteristics and porosity of the resulting vaterite particles.

When considering the observed change in the pore size of calcium carbonate obtained via a microfluidic chip, it is reasonable to infer that the union of multiple nuclei contributes significantly to this phenomenon. As particles come into contact with one another, their surfaces are likely to merge due to interfacial forces, thereby promoting an interconnected network of voids within the structure. This aggregation-driven growth mechanism subsequently shifts the distributions of pore sizes compared to those obtained from isolated or single-nucleus-based particle formation processes. It is important to note that the SAXS signal is observed from any fluctuations of electron density in the sample, and according to Babine’s principle [46], the intensity and shape of the intensity curve do not depend on the sign of the contrast. Therefore, it is impossible to distinguish between the scattering from the pores (which have a negative contrast relative to the average density) and from the matrix (particle) structure (with a positive contrast). In our case, the pores are supposed to have a more compact shape and higher contrast, so we assume that the shape of the intensity curve is mainly due to the scattering from the pore system.

The adhesion of several nuclei during particle formation enhances both the size and complexity of calcium carbonate structures, leading to an augmented pore size. The merging of multiple particles results in the creation of larger and interlinked internal voids within the final product. Consequently, this intricate network of interconnected pores offers notable benefits for various applications, such as enhanced mass transport, increased surface area, and improved drug delivery potential.

The adhesion mechanism plays a crucial role in the formation of calcium carbonate particles, as it facilitates the collision and subsequent binding of numerous nuclei. It has been widely documented that calcium carbonate nucleation occurs through the aggregation of ions and clusters, leading to the birth of primary particles. The propensity for these particles to adhere to one another during growth stages is primarily governed by intermolecular forces and local energy minima.

### 3.1. Loading Efficiency

The loading process was performed according to the well-defined procedure outlined in the Materials and Methods section. In this article, we employ the freezing technique to perform the loading of the particles obtained in the bulk phase. In general, this approach exhibits amplified efficiency in terms of loading, surpassing the co-precipitation method [10]. However, owing to the microfluidic device’s sole capability of facilitating loading exclusively through co-precipitation, we employed this method in our research. This procedure involved subjecting a suspension of particles to one or multiple cycles of freezing, whereby constant stirring was maintained along with the immersion of the particles in a solution of the target compound. In the present study, the targeted model compound employed for loading was a conjugate of the BSA-TRITC protein. According to the literature, the native BSA molecule has a nominal size of approximately 7 nm [55], which perfectly corresponds to the main pore size of vaterite particles (Figure 5b). 

To evaluate the loading capacity, a two-step process was employed. Initially, the vaterite particles were subjected to a loading procedure, during which they were exposed to the BSA-TRITC conjugate solution. The particles were then separated from the solution using centrifugation, and the resulting supernatant was collected for evaluation. Next, the release of the dye from the dissolved particles was assessed. The particles were dissolved, and the concertation of the released dye was analyzed using the spectrofluorometric method.

The analysis of the experimental data revealed significant differences in the loading capacity between the vaterite particles synthesized in the microfluidic chip and those precipitated in bulk. The loading capacity was found to be approximately 16 and 9 mass.% for the particles synthesized in a microfluidic device and in bulk, correspondingly. It should be noted that these results were obtained using an equal mass of particles (8 mg) for both types of synthesis. The disparity in loading capacity is likely attributed to the more developed surface and increased porosity as a result of the microfluidically assisted synthesis. BSA shows a natural tendency to dimerize, which increases its hydrodynamic size, and immobilization of BSA on the microfluidically derived vaterite ensures its existence mainly in an intact form due to the appropriate pore size. BSA loaded into vaterite particles produced in bulk synthesis is likely to be partly in native form and partly as dimers, and this cannot be controlled. These findings highlight the advantages of utilizing a microfluidic device for particle synthesis, particularly when aiming to achieve greater loading capacity. 

### 3.2. In Vitro Cytotoxicity

Another crucial aspect to consider in the study of synthesized submicron vaterite particles pertains to their potential cytotoxicity, particularly when intended for biomedical applications. Since the particles were synthesized within droplets in castor oil, concerns were raised that residual oil on the particle surface may contribute to increased cytotoxicity. Therefore, it becomes imperative to undertake an assessment of their cytotoxic effects in vitro. To address this concern, we conducted cytotoxicity evaluations on monolayers of mouse fibroblasts, using vaterite particles synthesized in the microfluidic chip as the focus of analysis, and vaterite synthesized in bulk as a control for comparison. The cells were exposed to varying concentrations of these particles, and their cytotoxic effects were studied using a standard viability test to estimate the cell viability. 

**Figure 6:**
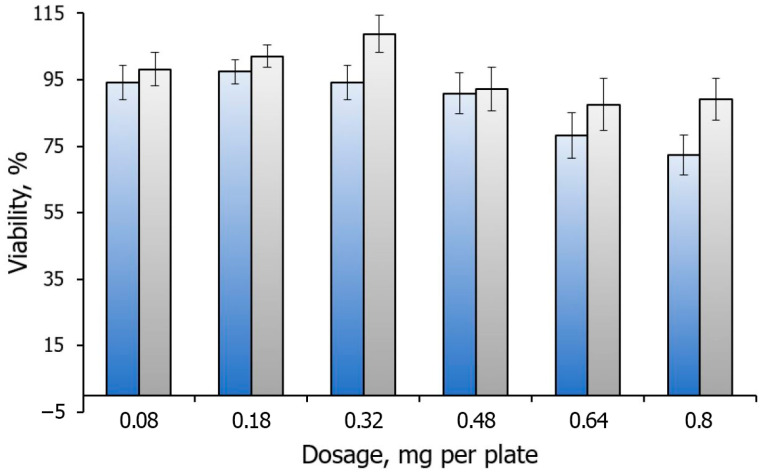
Viability profile of mouse fibroblasts after adding a dosage of control CaCO_3_ particles precipitated in bulk synthesis (grey bars) and after adding a dosage of CaCO_3_ synthesized via droplet-based approach in a microfluidic chip (blue bars).

The results obtained shed light on the potential toxicity of the synthesized particles and the suitability of their application in biomedicine. Remarkably, our findings indicate that the cytotoxicity of the microfluidically synthesized vaterite particles slightly exceeded the cytotoxicity observed in the control group (Figure 6). However, it is important to note that the increased toxic effects were observed only at a dosage surpassing 0.8 mg per plate. These results suggest that within a certain dosage range, the cytotoxic impact remains manageable. This result proves valuable for subsequent studies and necessitates further exploration to optimize the synthesis process and minimize any potential cytotoxic effects associated with residual oil. Understanding the cytotoxicity profile of these particles is pivotal in facilitating their safe application in biomedicine, where cellular interactions are of paramount importance. Further investigations and modifications can help refine the particle synthesis method to ensure a controlled and biocompatible formulation for future use in various biomedical applications.

Interestingly, our findings indicate a notable increase in cell viability at medium doses of calcium carbonate administered into the cellular milieu. The corresponding effect was previously shown by the authors of [56]. This observed phenomenon could potentially be attributed to the pivotal role calcium plays in cellular metabolic pathways. Conversely, at higher concentrations, a cytotoxic effect emerges, most likely arising from the mechanical perturbation of cellular membranes. Notably, when calcium carbonate was fabricated through microfluidic chip technology, the cytotoxic effect was marginally exacerbated, potentially attributable to the presence of oil residues on the material’s surface and the heightened reactivity of smaller particles. Thus, both enhanced interactions with cellular membranes and increased internalization into cells may contribute to this augmented cytotoxic response.

### 3.3. Outlook

The microfluidic synthesis of calcium carbonate enables enhanced control over the synthesis conditions, as it maximizes the surface-to-volume ratio and provides precise control of the reactant resource. Furthermore, it paves the way for scaling up the synthesis process. This study and the extensive literature highlight the synthesis capability of approximately 8 mg of submicron calcium carbonate at a time, requiring a reaction time of one and a half to two and a half hours, followed by lengthy solvent washing. In contrast, the utilization of a microfluidic chip allows for the synthesis of calcium carbonate at a rate ranging from 20 to 80 mg per hour. Nonetheless, this method encounters certain challenges, such as increased particle aggregation when reducing their average size, as observed in this investigation. The high aggregation tendency observed in the particles synthesized in the microfluidic device can arise due to excess free energy within the system and the merging of droplets during the synthesis process. To mitigate these challenges, the addition of surfactants to the oil can help stabilize the droplets, while an additional polymer can be introduced into the particle suspension to charge the particle surface and mitigate aggregation. However, it is important to consider the potential consequences associated with using surfactants and additional polymer layers. Surfactants such as span80 are effective in stabilizing droplets by reducing interfacial tension and preventing coalescence [57]. Nevertheless, they may also introduce an increased level of toxicity when present on the particle surface. These surfactants, commonly amphiphilic in nature, can interact with the surrounding cellular environment and potentially cause adverse effects. Therefore, careful consideration is required when selecting surfactants to ensure their suitability for biomedical applications. Alternatively, an additional layer of polymer can be applied to the particle surface to promote stability and avoid aggregation. This layer can contribute to charging the surface and repelling particles from one another. However, the addition of an extra polymer layer may hinder the subsequent loading of target substances onto the particles. Consequently, it becomes necessary to optimize the loading process to overcome any undesired disruptions caused by this additional polymer layer. While the utilization of surfactants and additional polymer layers offer valuable strategies in addressing aggregation and stability concerns during particle synthesis, their implementation necessitates thorough examination and optimization. The potential toxicity introduced by surfactants necessitates the careful selection of appropriate surfactants that exhibit minimal adverse effects. Similarly, the impact of an additional polymer layer on the substance-loading process should be considered, leading to the requirement of conducting the loading process prior to the application of an extra polymer layer. Further research must focus on refining these strategies to attain ideal particle stability without compromising their biocompatibility or loading capabilities. Alternative methods to address particle aggregation, such as adjusting synthesis conditions or exploring different surfactant types, should be explored to enable optimized particle fabrication without compromising their intended applications in biomedicine.

## 4. Conclusions

This study presents an approach for synthesizing calcium carbonate particles in the form of vaterite using a microfluidic device with a T-shaped oil supply system. Characterization through SEM, DLS, Raman spectroscopy, and XRD revealed that the particles produced using the microfluidic device display reduced size (from an average hydrodynamic diameter of 600 nm down to an average of 100 nm) while keeping the fraction of calcite at a low level (in the range of 3–13%). Furthermore, the high rate of particle synthesis achieved through the microfluidic device offers advantages such as scalability and reproducibility. Our findings reveal that the acquired particles exhibited a notable enhancement in loading capacity by approximately 16 mass%. This increased loading efficiency can be attributed to two contributing factors: firstly, the smaller particle size enabled a larger amount of material to be accommodated within the particles. Secondly, the concurrent development of the particle surface allowed for a more substantial surface area, facilitating greater loading efficiency. Additionally, the main fraction of pore sizes in the range of 5–10 nm was observed by SAXS for the particles precipitated in the confined microvolumes and in bulk. As the vaterite particles precipitated in the microvolumes have no larger pores, unlike another sample, we can be confident that there are no protein dimers among the immobilized molecules. These results collectively highlight the potential of the obtained particles to serve as highly efficient carriers for various applications, underscoring the importance of size and surface characteristics in enhancing loading capacity. In vitro studies have indicated a negligible increase in cytotoxicity for these particles. Through the in vitro assessments using mouse fibroblast monolayers, we observed that the cytotoxicity of vaterite particles synthesized in the microfluidic chip slightly surpassed the control, becoming toxic only at dosages exceeding 0.8 mg per plate. These findings emphasize the importance of careful optimization and validation of particle synthesis techniques to minimize potential cytotoxic effects and ensure the safe application of such particles in biomedicine.

## Data Availability

Data are available on request from the corresponding authors. The data are not publicly available due to privacy issues.

## Supplementary Materials

The following supporting information can be downloaded at https://www.mdpi.com/article/10.3390/mi15010016/s1. Figure S1: Raman spectrum of calcium carbonate in the form of vaterite (a), combination of calcite and vaterite (b), and strong calcite peak (c); Figure S2. XRD spectra of calcium carbonate particles obtained in the bulk conditions (a) and using microfluidic device (b).
