# Supplementary figures and images for: Microfluidically Assisted Synthesis of Calcium Carbonate Submicron Particles with Improved Loading Properties
Source: Micromachines (Basel). 2023 Dec 21;15(1):16. doi: 10.3390/mi15010016 (PMC10818696; doi:10.3390/mi15010016)

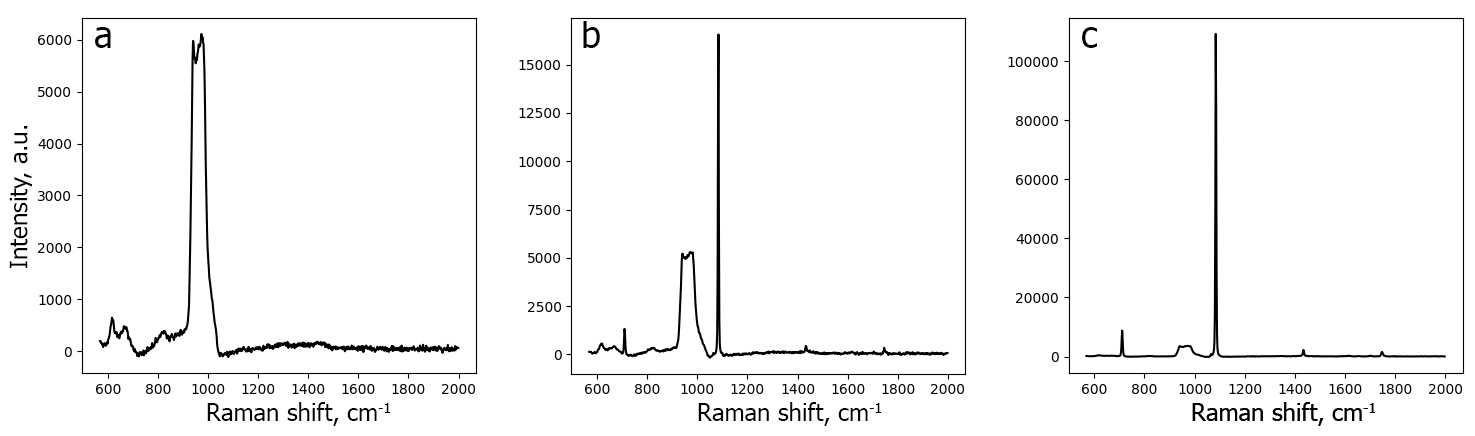

Supplement: Supplementary file 1 [file micromachines-15-00016-s001.zip › Figure S1 Raman spectrum of calcium carbonate in the form of vaterite (a), combination of calcite and vaterite (b), and strong calcite peak (c).tif]

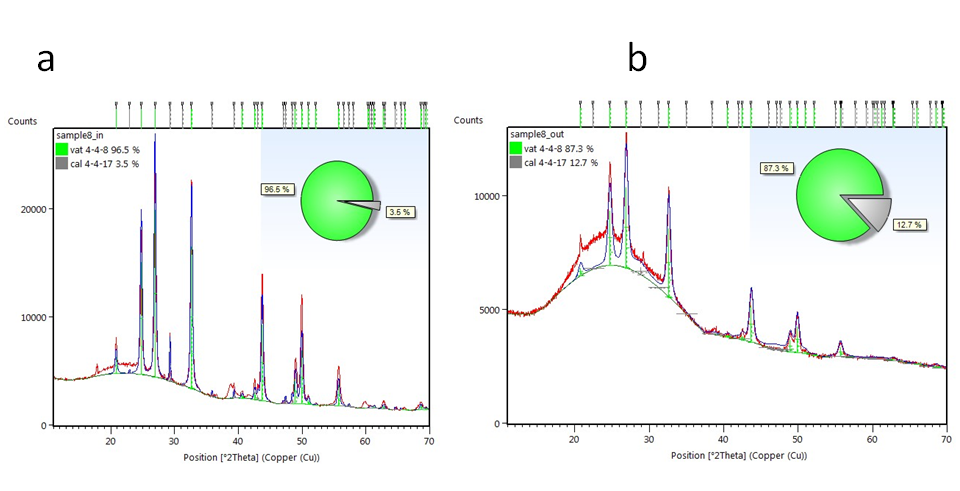

Supplement: Supplementary file 1 [file micromachines-15-00016-s001.zip › Figure S2. XRD spectra of calcium carbonate particles obtained in the bulk conditions (a) and us-ing microfluidic device (b).tif]
